# Hope, optimism and delusion

**DOI:** 10.1192/pb.bp.113.044438

**Published:** 2014-04

**Authors:** Rebecca McGuire-Snieckus

**Affiliations:** 1 Department of Psychology, Bath Spa University

## Abstract

Optimism is generally accepted by psychiatrists, psychologists and other caring professionals as a feature of mental health. Interventions typically rely on cognitive-behavioural tools to encourage individuals to ‘stop negative thought cycles’ and to ‘challenge unhelpful thoughts’. However, evidence suggests that most individuals have persistent biases of optimism and that excessive optimism is not conducive to mental health. How helpful is it to facilitate optimism in individuals who are likely to exhibit biases of optimism already? By locating the cause of distress at the individual level and ‘unhelpful’ cognitions, does this minimise wider systemic social and economic influences on mental health?

Optimism is generally accepted by psychiatrists, psychologists and other mental health professionals as a preferred way of being. ‘Hope and optimism about the future’ was identified as one of five processes for recovery in mental illness in a systematic review and narrative synthesis of 97 papers in psychiatry.^1^ Hope (including optimism) is classified as a ‘character strength’ in the ‘manual of the sanities’ - *Character Strengths and Virtues* - which was written by positive psychologists in response to the DSM and which identifies key ‘strengths’ that elevate an individual from ‘suboptimal’ to ‘optimal’ functioning.^2^ Cognitive-behavioural therapy is offered on the National Health Service (NHS) to ‘help you change your negative thought patterns and improve the way you feel’.^3^ Indeed, a position of optimism for the NHS system as a whole was called for by the chief executive Mike Farrar at the 2013 NHS Confederation Conference with his claim that ‘If we’re not optimistic for the future, then we deserve to fail.’^4^

## Early contexts of hope and optimism

Although used as interchangeable concepts by some and as mutually exclusive concepts by others,^5^ the term hope precedes optimism. In classical antiquity, hope was the last of the wedding gifts Zeus gave to Epimetheus and his wife, Pandora, really a punishment to the groom and his brother, Prometheus, for giving mortal man fire. When opened by the new bride Pandora, the ills of humanity were unleashed, while hope, blind, fluttered at the lip of the jar.^6^ Nietzsche interprets hope in this context as ‘the most evil of evils because it prolongs man’s torment’.^7^ In the English language, the word hope found its earliest use in the context of forlorn hope. It derives from the Dutch military expression *verloren hoop* - referring to lost troops. *Hoop* was mistaken for hope and ‘the phrase came to mean a body of desperate men who have abandoned all hope for surviving, a desperate enterprise, as in to cherish a forlorn hope’.^8^ In Judeo-Christianity, hope is among the ‘greatest of all gifts’, along with faith and charity in 1 Corintheans 13. Hope is defined by some psychologists as ‘the perceived capacity to derive pathways to desired goals, and motivate oneself via agency thinking to use those pathways.’^9^

The term optimism was not used until 1710 by Leibniz in his work *Théodicée* to mean the greatest good (derived from *optimus* in Latin) by suggesting that good will ultimately prevail over evil. In his novel *Candide*, Voltaire was derisive of the shallowness of an optimistic worldview. According to William James, ‘pessimism leads to weakness, optimism to power.’^10^ Some psychologists today regard optimism as a disposition characterised by positive expectation,^11^ whereas others define it as an explanatory style characterised by a tendency to attribute negative outcomes to external causes, specific and temporary causes and positive outcomes to internal, global and permanent causes.^12^ The construct as an explanatory style was given popular force by Martin Seligman, credited as the ‘father of positive psychology’, whose research focus turned from the study of ‘learned helplessness’ in dogs as a model for depression in humans (by demonstrating that dogs who were persistently shocked without the opportunity to escape did not later perceive opportunities to escape, as they had ‘learned’ to be ‘helpless’) to ‘learned optimism’. He observed that some dogs never learned to be helpless and began investigating why some people do not give up after being exposed to repeated stressors.^12^

## Optimism and individualism

Optimism is viewed by many as an indication of mental health, associated with higher levels of subjective well-being, better physical health and more success.^13^ Some propose optimists provide ‘models of living for others to learn from’, citing findings that link optimism to better subjective well-being, better physical health, persistent educational efforts, higher income and better relationships.^14^ As much of the evidence is based on correlational research which cannot infer causation, it could be equally argued that better health, education, income and good relationships could be predictive of an optimistic worldview, and not the reverse. Indeed, the same authors concede that ‘a poor childhood socioeconomic circumstance breeds pessimism later in life’.^14^

It could be argued that much discourse around optimism may be a function of individualism. By identifying distress as a ‘psychological matter’ rather than locating it in ‘the social and material world with which we are intimately interconnected’,^15^ key proponents ‘promote positive thinking and to systematically dispel the negative thoughts that affect us all’^16^ despite epidemiological evidence that fewer economic and social resources predict higher mental and physical health problems throughout life.^17^ It has been further argued that ‘the current official preoccupation with happiness’ may be at best ‘a naïve attempt to improve the world through wishful thinking, and at worst a form of insidious social control, where people are encouraged to look inwards for the sources of their troubles, and in the end to implicitly blame themselves for these ills’.^15^

## Interventions that improve optimism

Interventions are used to increase optimism in both clinical and non-clinical populations. Cognitive-behavioural therapy is offered on the NHS to stop ‘negative thought cycles’ based on the presupposition that ‘there are helpful and unhelpful ways of reacting to a situation, often determined by how you think about them’.^3^ Such ‘cognitive restructuring tools’ which encourage individuals to ‘challenge unhelpful thoughts’ that threaten self-esteem and therefore mental health are used to prevent mental health problems, to enhance performance and to reduce work-related stress.^18^ Some interventions emphasise the importance of ‘increasing the frequency of positive cognitions and self-statements that foster optimism’ to combat ‘everyday malady’;^19^ whereas others encourage clients to adopt an ‘as if’ approach to life, to ask clients to ‘pretend that their lives are improved in some way, however small, and to experience their lives anew in the face of these positive changes’.^20^ Blackwell *et al* propose that ‘boosting positive future imagery’ to promote optimism could provide ‘implications for mental health and even physical well-being’.^21^ Further interventions to improve optimism encourage individuals to make external attributions for negative outcomes and internal attributions for positive ones to improve success.^22^

## Cognitive biases

However, social psychological research reveals that most individuals already tend to exhibit persistent cognitive biases characterised by biases in probabilistic reasoning and attribution.^23^ The ‘optimism bias’ shows that when asked to make comparative judgements about future life events, individuals consistently expect that positive events are more likely to happen to them (i.e. having a gifted child) and that negative events (i.e. divorced after a few years) are less likely.^24^ The ‘better than average effect’ demonstrates that individuals tend to evaluate themselves more favourably compared with others.^25^ A variety of biases of attribution have also been identified, from the self-serving bias (the tendency to deny responsibility for failure and take credit for success) and the self-centred bias (taking more credit for a jointly produced outcome) to the false consensus effect (the tendency to see one’s own behaviour, thoughts and feelings as typical).^26^ Other self-favouring biases have been identified such as spatial biases (things are better here than there), environmental comparative optimism (things are environmentally safer here than elsewhere) and temporal biases (discounting the importance of a problem the farther away in the future that it seems).^27^

### Delusions

Delusions are characterised by biases in attribution and probabilistic reasoning.^28-30^ Karl Jaspers viewed delusions as deeply-held beliefs that are impervious to logic.^31^ If not slightly deluded, the average individual does appear to be consistently biased. Perhaps, as Festinger *et al* suggested, we are not rational, but ‘rationalising’ animals.^32^

### Depressive realism

Biases of optimism are said to apply to most people, but generally not to individuals with depression (termed depressive realism). Although some have found no support for the notion of depressive realism but rather suggest that patients with depression ‘distort their judgements in a characteristically negative fashion’,^33^ there appears to be reliable evidence that even individuals with depression exhibit persistent cognitive biases of optimism.^34^ A meta-analytic review of 75 studies representing 7305 individuals indicated a small overall effect of depressive realism (Cohen’s *d* = –0.07) and that both individuals with depression (*d* = 0.14) and individuals without depression (*d* = 0.29) showed a substantial positive bias.^34^

## Excessive optimism

Although optimism may serve the function to motivate individuals in the present in the service of future goals, excessive optimism may blind individuals to perceive the inherent risk in their present actions, resulting in consequences that individuals might better permit themselves to anticipate. Optimism is credited with predicting a catalogue of negative outcomes from risk-taking in unprotected sex,^35^ underestimating risks in driving,^36^ continued gambling after losses,^37^ engagement in music piracy,^38^ to minimising the health consequences of smoking.^39^ Moreover, optimism can create the potential for unmet expectations and heightened negative reactions when such expectations are not realised, including increased physical and psychological symptoms and reduced mental health.^40,41^

More clarity is needed regarding this concept. What is the purpose of facilitating biases of optimism in a non-clinical population who is likely to have persistent esteem-enhancing biases of attribution and optimism already? If excessive optimism can lead to a catalogue of ills, how are the proposed techniques designed to facilitate optimism conducive to mental health in a non-clinical population? How helpful are such interventions even among individuals with depression who appear to also exhibit persistent cognitive biases of optimism?^34^ By locating the cause of optimal or suboptimal functioning at the individual level, does this minimise wider systemic social and economic influences on mental health?
